# Tracheobronchomegaly associated with tracheobronchopathia osteochondroplastica: a case report

**DOI:** 10.3389/fmed.2024.1444995

**Published:** 2024-09-27

**Authors:** Zhen Hua Li, Lu-Xia Kong, Shan Zhu, Yi Hu, Shan Gao

**Affiliations:** Department of Respiratory and Critical Care Medicine, The Central Hospital of Wuhan, Tongji Medical College, Huazhong University of Science and Technology, Wuhan, China

**Keywords:** tracheobronchomegaly, Mounier-Kuhn syndrome, tracheobronchopathia osteochondroplastica, recurrent pneumonia, case report

## Abstract

Tracheobronchomegaly (TBM) is a rare condition characterized by the dilatation of the trachea and bronchi due to severe atrophy of elastic fibers, accompanied by the thinning of the muscularis mucosae and the development of diverticula between cartilaginous rings. The etiology of this condition remains unclear. Tracheobronchopathia osteochondroplastica (TO) is another uncommon airway disease with an unknown etiology. The co-occurrence of these two diseases has not been reported. In this study, we report and discuss a case involving an elderly man with TBM and TO with a history of recurrent pneumonia over the past 6 years.

## Introduction

Tracheobronchomegaly (TBM), also known as Mounier-Kuhn syndrome (MKS), is different from other morphological abnormalities of the central airways (1). Tracheobronchopathia osteochondroplastica (TO) is another uncommon airway disease with an unknown etiology (2). This report presents an exceptional case of TBM in an adult, which can be attributed to TO. An 84-year-old man with a history of recurrent pneumonia in the same regions for the past 6 years underwent a chest computed tomography (CT) scan and fibroscopic examination, which revealed TO as the underlying cause of his condition. To the best of our knowledge, this is the firstdocumented case of adult TBM specifically associated with TO involving the trachea. The potential predictors of TBM and TO remain unclear. The diagnosis of this condition is challenging due to the presence of overlapping symptoms and a lack of awareness, often resulting in missed identification and unnecessary medical interventions.

## Case description

**Figure 1:**
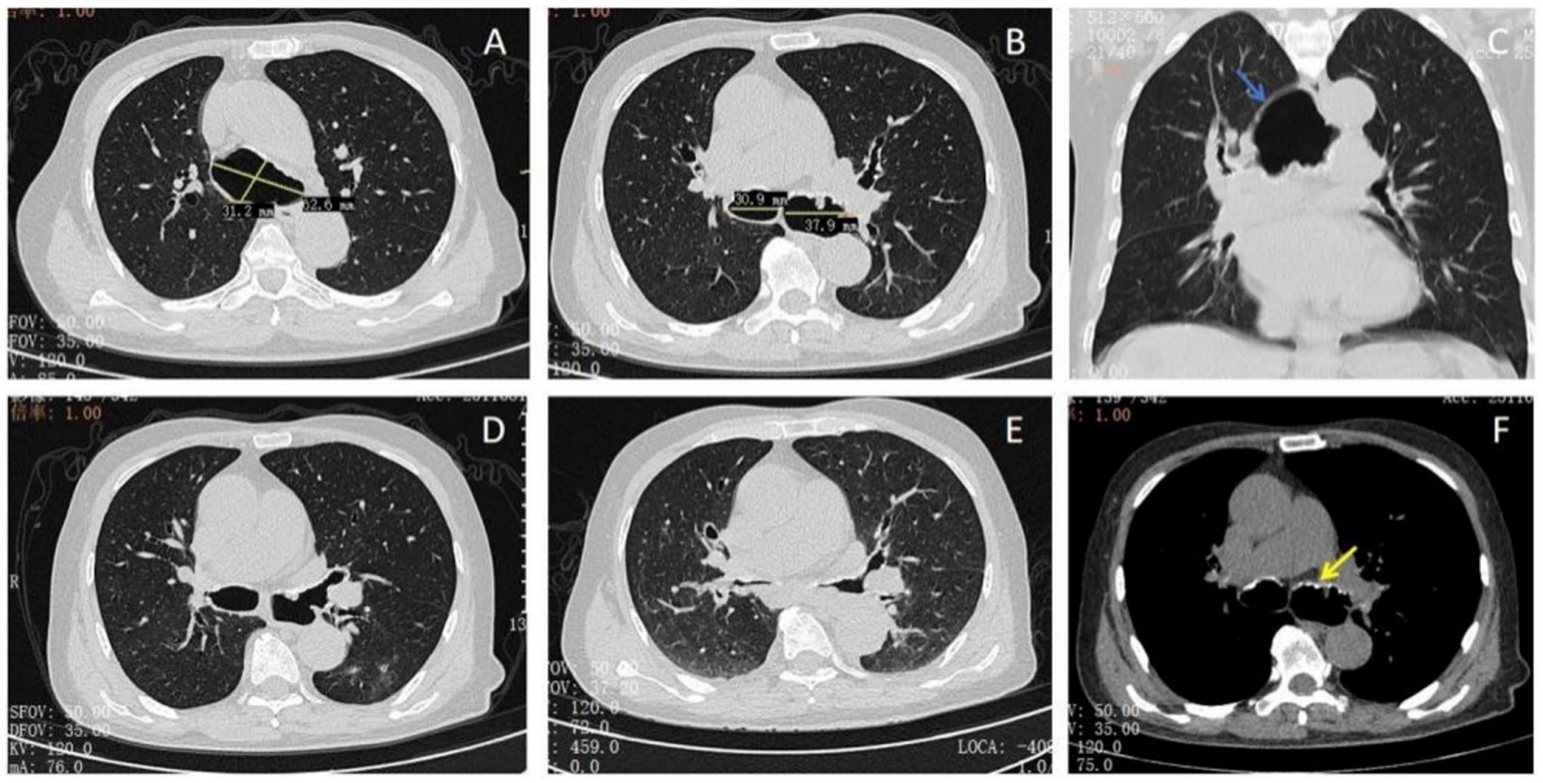
Thoracic computed tomography: **(A)** dilation of the trachea (62.6*31.2 mm). **(B)** Dilation of the main bronchi shown above. **(C)** The muscular layer of the tracheal wall was evidently thinner (blue arrow). **(D,E)** The constriction of the main bronchi was more than 50% during expiration. **(F)** CT images showed irregular submucosal nodular calcification (yellow arrow).

**Figure 2:**
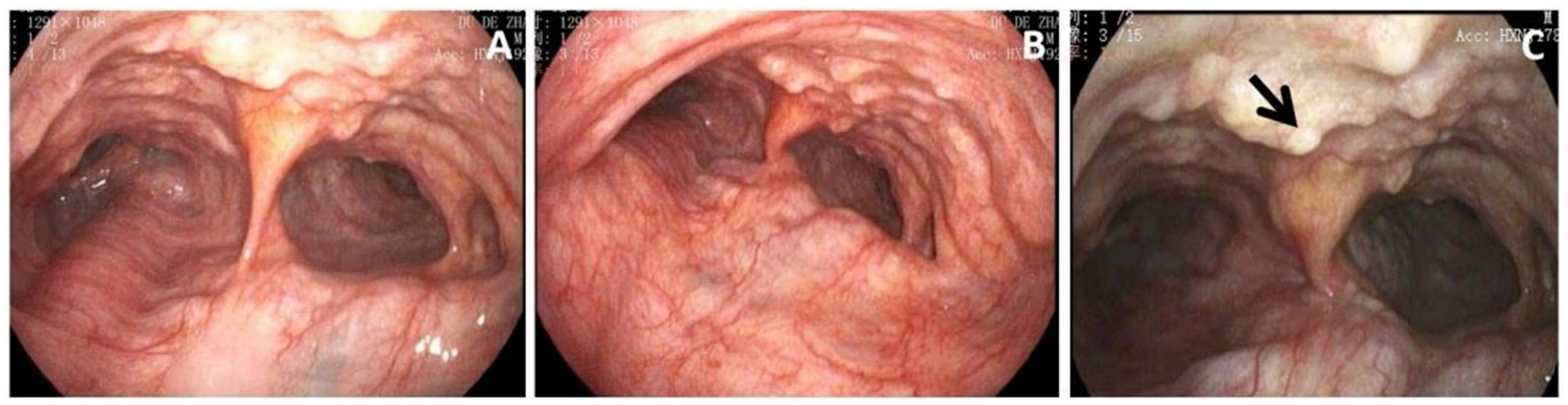
**(A)** Bronchoscopy showed massively dilated trachea. **(B)** The constriction of the main bronchi was approximately 50% during expiration. **(C)** Numerous cartilaginous and bony nodules protruding into the lumen from the submucosa of the tracheobronchial tree (black arrow). Histopathological examination of these lesions was performed to exclude neoplastic or chronic granulomatous etiologies. The results were suggestive of nodular cartilaginous tissue and mature bone tissue with calcification, as shown in Figure 3.

**Figure 3:**
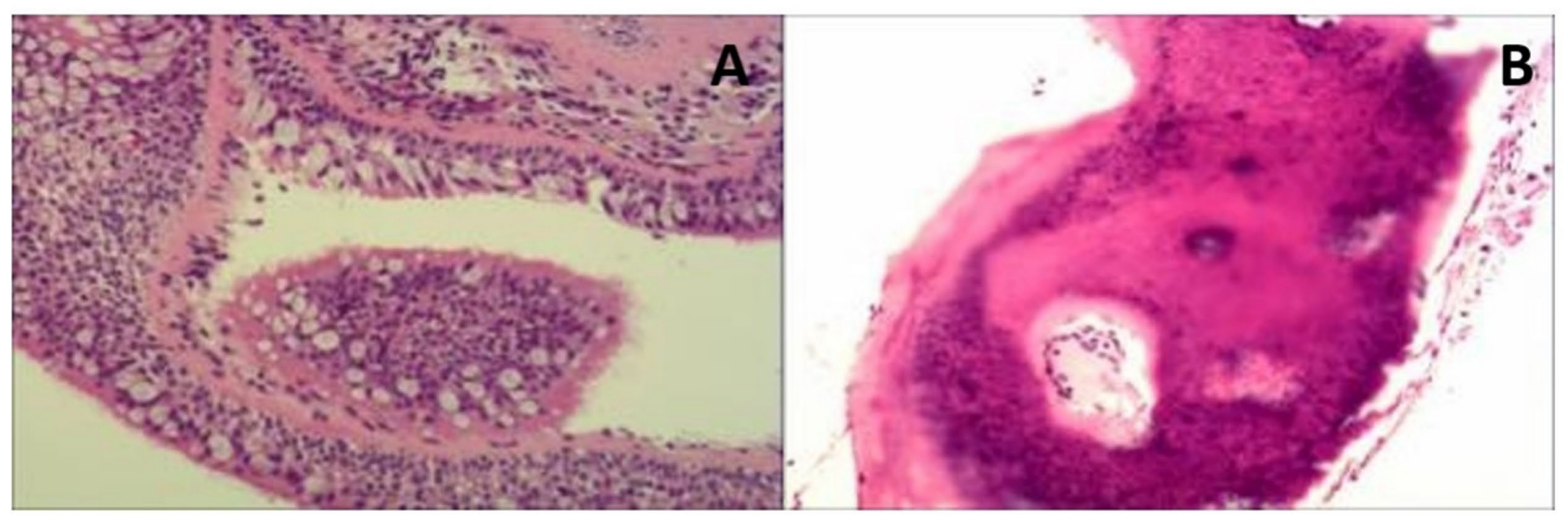
Histopathological images (biopsy taken during the bronchoscopy). **(A)** The bronchial mucous membrane tissue showed chronic inflammatory changes. **(B)** There was some calcified bone tissue in the local area. The patient was managed conservatively with close observation, demonstrating a gradual progression course over a 6-year follow-up period. Symptoms during each hospitalization period were alleviated through the administration of antibiotics.

The patient, an 84-year-old retired worker, presented with a 6-year history of chronic cough accompanied by mucopurulent expectoration. The onset of fever occurred the day before he was presented. He has a past medical history of recurrent pneumonia, requiring multiple hospitalizations. He is currently a non-smoker but had a 10-year history of smoking 20 cigarettes per day. In addition, the patient has a long-standing diagnosis of hyperlipidemia and is on atorvastatin calcium medication. His familial medical history only includes arterial hypertension in his father. A computed tomography (CT) scan of the chest revealed tracheomegaly with a diameter of 62.6 mm and an increased caliber of the great bronchi (30.9 mm on the right and 37.9 mm on the left) (Figures 1A–E). The muscular layer of the tracheal wall was evidently thinner (Figures 1A–E), which is characterized by atrophy or the absence of elastic fibers or smooth muscle in the wall of the trachea and the main bronchi. A slice acquired during expiration demonstrated a partial collapse of the trachea and main bronchi (Figures 1A–E). The chest CT scan revealed an irregular calcified appearance of the tracheal wall and both main bronchi (Figure 1F). The video bronchoscopy revealed dynamic partial stenosis during expiration, indicating tracheomalacia (Figure 2). During the bronchoscopy, the increased tracheal diameter and the expiratory collapse due to tracheomalacia were observed, and the redundant tracheal wall might have even obstructed the view, which was consistent with the CT scan (Figure 3).

## Discussion

Tracheobronchomegaly (TBM), first documented in 1932, is a rare and most likely congenital syndrome characterized by an enlarged trachea and main bronchi (3). To date, fewer than 400 cases have been documented in medical literature. TBM is distinguished by the dilation of the trachea and bronchi, setting it apart from other morphological abnormalities of the central airways. Its etiology remains unknown. However, it is considered to involve familial susceptibility and inheritance through autosomal recessive mechanisms, including Ehlers–Danlos syndrome, cystic fibrosis, Marfan’s syndrome, and ankylosing spondylitis (4). The diagnosis of TBM is established through bronchoscopy and radiological imaging techniques. The use of CT has enabled better and easier visualization and determination of the airway size, facilitating the recognition of evidently abnormal cases. The tracheal diameter exceeding 3 cm or mainstem bronchi diameters greater than 2.4 cm strongly indicates the presence of the disease. For our patient, the transverse diameter of the trachea was 62.6 mm, the anteroposterior diameter was 31.2 mm, the diameter of the left main bronchus was 37.9 mm, and the diameter of the right main bronchus was 30.9 mm. A biopsy and necropsy examination of the tracheal wall revealed thinning of the muscularis mucosae, accompanied by the loss of elastic fibers (5). The effect of enlarged airways on spirometry derives from the weakness of the tracheobronchial walls and hypotonia in the myoelastic elements, resulting in dynamic airway compression (manifesting as an expiratory collapse during forced exhalation) and dynamic restriction. The clinical features are non-specific. Some patients remain asymptomatic with normal respiratory function, while others exhibit chronic cough and recurrent lower respiratory tract infections, which ultimately progress rapidly to severe respiratory failure and mortality (6). However, the etiology remains unclear and requires further follow-up. The condition known as tracheobronchopathia osteochondroplastica (TO) was initially documented by Rokitanski in 1855 (7). It is a rare, idiopathic, and benign disease that is often underdiagnosed. The reported incidence of TO is 0. 11% (8). With the advancement of research and the widespread utilization of bronchoscopy, an increasing number of reports have been documented. Currently, there are over 600 recorded cases of TO (9). The condition is characterized by the presence of multiple submucosal nodules composed of cartilage and bone in the anterolateral walls of the tracheobronchial tree, with the exception of the posterior wall (10). The difficulty in clamping bronchoscopic nodules for a biopsy renders it unnecessary to perform biopsies in all cases (11). However, it is crucial to differentiate the histomorphology of TO from calcification resulting from other conditions such as tracheobronchial amyloidosis, atrophic polychondritis, and granulomatous diseases, such as TB or sarcoidosis. The pathogenesis of TO remains elusive, with speculations suggesting that it may arise from submucosal elastic fibrosis, leading to the formation of elastic cartilage, which subsequently undergoes calcification and ossification. Alternatively, it is proposed that TO could result from the excessive proliferation of tracheal cartilage rings, giving rise to exophytic osteochondral warts that eventually ossify (12). The presence of TO has been found to be associated with certain malignancies, such as skin cancer and polyarteritis nodosa, as well as IgA deficiency. However, there is currently no established pathophysiological correlation between TO and these diseases (13, 14). The clinical manifestations are non-specific and resemble those of TBM, which include cough, recurrent respiratory infections, and hemoptysis (15). During progression to a severe stage, the nodules protrude into the lumen of the trachea and main bronchi, potentially causing airway obstruction. In our case report, we observed significant expansion of the main bronchi, which has not been previously documented. There is currently no clear pathological association between TBM and TO. The exploration of this issue requires further investigation. In our case report, we assumed that the ossified bronchial stenosis in the right lower lung potentially resulted in the weakening of the tracheal wall. The chronic high pressure generated by the stenosis during expiration most likely led to the gradual dilation of the trachea. More case reports are needed to confirm this hypothesis. The present case report highlights the importance of CT and fiberscopic examination for the diagnosis and shows that TBM can be associated with TO. Currently, for patients with tracheal stenosis caused by osteocartilaginous nodules, non-invasive continuous positive airway pressure ventilation, bi-level positive airway pressure ventilation, invasive stent placement, and surgical treatment are mainly used (16). However, while positive airway pressure ventilation can only temporarily relieve clinical symptoms, it cannot serve the purpose of treating the disease. Stent implantation is often associated with complications such as secretion retention, infection, and restenosis due to granulation. Surgical treatment causes significant damage to patients, involves high risks and complications, and has certain limitations. With increasing clinical awareness, it is essential to identify clinical risk factors and improve treatment outcomes. Laser tracheobronchoplasty is one of the less invasive new techniques. Laser tracheobronchoplasty treatment can reduce local nodule contracture and the formation of scar, thereby improving airway collapse. Currently, domestic and foreign studies have reported the effect of this treatment with good clinical feedback, and the clinical symptoms of patients have been reported to significantly improve within 1 week after the surgery (17). Therefore, laser tracheobronchoplasty is a safe and effective technique for the treatment of osteocartilaginous nodules to achieve recanalization. However, future studies regarding the effectiveness of this technique are needed.

## Data Availability

The original contributions presented in the study are included in the article/supplementary material, further inquiries can be directed to the corresponding author.
