# Corrigendum: Tracheobronchomegaly associated with tracheobronchopathia osteochondroplastica: a case report

**DOI:** 10.3389/fmed.2024.1540232

**Published:** 2024-12-17

**Authors:** Zhen Hua Li, Lu-Xia Kong, Shan Zhu, Yi Hu, Shan Gao

**Affiliations:** Department of Respiratory and Critical Care Medicine, The Central Hospital of Wuhan, Tongji Medical College, Huazhong University of Science and Technology, Wuhan, China

**Keywords:** tracheobronchomegaly, Mounier-Kuhn syndrome, tracheobronchopathia osteochondroplastica, recurrent pneumonia, case report

In the published article, there was an error in affiliation. Instead of “^1^The Centre Hospital of Wuhan, Wuhan, China, ^2^Huazhong University of Science and Technology, Wuhan, China”, it should be “Department of Respiratory and Critical Care Medicine, The Central Hospital of Wuhan, Tongji Medical College, Huazhong University of Science and Technology, Wuhan, China”.

The authors apologize for this error and state that this does not change the scientific conclusions of the article in any way. The original article has been updated.

